# Editorial: Microbial-fungal symbioses: ecological implications, environmental impact, and biotechnological applications in natural and agricultural systems

**DOI:** 10.3389/fmicb.2026.1861156

**Published:** 2026-05-12

**Authors:** Hector Herrera, Amit Sinha, Soumyadev Sarkar, Juan Carlos Cambronero-Heinrichs

**Affiliations:** 1 Center for Biodiversity and Ecological Sustainability (C-BEST), Facultad de Ciencias Agropecuarias y Medioambiente, Universidad de La Frontera, Temuco, Chile; 2 New England Biolabs, Ipswich, MA, United States; 3 Center for Fundamental and Applied Microbiomics, Biodesign Institute, Arizona State University, Tempe, AZ, United States; 4 Programa de Investigación en Enfermedades Tropicales (PIET), Escuela de Medicina Veterinaria, Universidad Nacional de Costa Rica, Heredia, Costa Rica; 5 Centro Nacional de Innovaciones Biotecnológicas (CENIBiot), Centro Nacional de Alta Tecnología (CeNAT)-Consejo Nacional De Rectores (CONARE), San Josè, Costa Rica

**Keywords:** bioinoculants, chemical disturbance responses, endohyphal bacteria, hyphosphere microbiome, metabolome dynamics, microbiome assembly drivers

Microbial-fungal symbioses are fundamental drivers of ecological networks and agricultural systems, orchestrating critical processes such as nutrient cycling, maintenance of soil and plant health, and enhancement of host resilience across diverse environments (Martin and van der Heijden, 2024; Williams et al., 2023). These interactions, spanning a continuum from mutualism to commensalism and antagonism, involve fungi engaging with taxonomically diverse partners (Martin and Tan, 2025). Under the pressures of global environmental change, land degradation, and intensifying agricultural demands, understanding the mechanistic basis and ecological consequences of these interactions has become increasingly urgent and a clear research priority.

This Research Topic aims to consolidate pioneering research focused on the mechanistic basis and diversity of microbial-fungal partnerships, addressing key questions related to the molecular and ecological mechanisms that regulate microbiome-host interactions. The 12 publications included in this Research Topic have expanded our understanding of the role of these symbioses across diverse contexts, including medicinal plants, fruit crops, hyphosphere-associated bacteria, and rhizosphere and endophytic interactions.

Fungal endophytes modulate plant fitness through tissue-specific colonization, thereby influencing stress tolerance and secondary metabolite production. Elgar et al. investigated the endophytic colonization of *Prunus persica* seedlings by the entomopathogenic fungus *Beauveria bassiana*. The authors confirmed systemic fungal colonization across leaf, stem, and root tissues and quantified beauvericin (BEA), which was detected mainly in non-surface-sterilized tissues. Foliar-sprayed leaves showed the highest BEA concentrations, indicating a predominantly epiphytic origin of metabolite production. Liu et al. examined the metabolite diversity of the fungal endophyte *Epichloë sinensis* isolated from *Festuca sinensis*. Their results demonstrated that *E. sinensis* harbors a rich secondary metabolome, which is strongly influenced by the culture medium. Arcela-Castro et al. analyzed endophytes associated with marine macroalgae from the northern Peruvian coast. The authors identified a strain of *Aspergillus sydowii* with plant growth-promoting traits, highlighting their potential for enhancing maize growth under saline stress. Collectively, these case studies demonstrate that microbial-promoted benefits depend on spatial colonization and environmental context.

Bacterial-fungal consortia constitute critical determinants of microbiome functionality and host resilience, playing essential ecological roles that often result in enhanced stress tolerance and increased functionality in the soil microbiome. Liang et al. analyzed the diversity of endohyphal bacteria associated with oil-producing fungi. In their analyses, the authors detected Proteobacteria as principal endohyphal bacteria in oil-producing fungal isolates. Zhou, Pan et al. investigated how intercropping reshapes the bacterial communities associated with arbuscular mycorrhizal fungal spores in the tomato rhizosphere. The authors showed that intercropping increased alpha diversity and significantly altered community composition compared to monoculture systems, enriching taxa such as *Streptomyces, Rhodococcus, Paenarthrobacter*, and *Janthinobacterium*. Zhou, Liu et al. investigated the interaction between the medicinal fungus *Polyporus umbellatus*, its obligate symbiont *Armillaria gallica*, and the rhizosphere bacterial community. The authors demonstrated that the co-presence of both fungi increased bacterial operational taxonomic unit richness and diversity indices, with Proteobacteria, Acidobacteriota, and Bacteroidota being enriched, where an isolate of *Rhodococcus* sp. was defined as a potential bioinoculant to enhance medicinal fungus cultivation. Together, these studies demonstrate that bacteria-fungus associations can enhance stress tolerance, nutrient acquisition, and cultivation efficiency.

Beyond their well-known roles in plant growth promotion, microorganisms also influence plant metabolic regulation and phenotype expression. Xie et al. studied the succession of the endophytic microbiome and metabolome dynamics across four ripening stages of the medicinal plant *Rubus chingii* Hu. In their study, they demonstrated that bacterial and fungal communities were positively correlated with the accumulation of key secondary metabolites such as ellagic acid, flavonoids, and phenolic acids. Qin et al. investigated phenotypic traits of *Rosa roxburghii* across environmentally heterogeneous sites and characterized rhizosphere bacterial and fungal communities. Their results demonstrated that fungal markers exhibited broader associations with phenotypic traits than bacterial markers, and regression analyses indicated that fungal markers were statistically associated with variation in fruit size. Shen et al. examined the synergistic potential of combining biochar amendment with halophyte intercropping (*Paspalum vaginatum*) as a strategy to mitigate soil constraints related to continuous cucumber cropping in greenhouse systems. The authors demonstrated that the combined treatment improved soil parameters, resulting in increased yield and fruit quality, as well as an increase in the soil bacterial diversity. Wang et al. characterized the soil fungal community structure of *Leucocalocybe mongolica*. The authors demonstrated that the fruiting zone showed reduced alpha diversity, simplified microbial networks, and markedly elevated laccase activity, with saprotrophic guilds dominating in contrast to the ectomycorrhizal-dominated peripheral zones. Collectively, these studies highlight the dynamic interplay between microbial communities, environmental factors, and plant metabolic and phenotypic outcomes.

Microbial communities exhibit dynamic responses to chemical disturbances and external inputs, with important consequences for ecosystem stability and function. Qu et al. provide comprehensive insights into the recovery of soil microbial communities in degraded coal mine dumps. The results reveal that bacterial communities exhibit strong resilience and structural complexity during restoration, whereas fungal communities remain persistently constrained. Yang et al. investigated how the insecticide tetraniliprole and the biopesticide *Metarhizium anisopliae* affect the gut fungal community structure of the predatory insect *Arma custos*. The authors demonstrated that *M. anisopliae* colonization shifts the gut fungal community, mainly suppressing fungal diversity, while tetraniliprole promotes opportunistic taxa such as *Candida parapsilosis* and *Aspergillus penicillioides*. Together, these studies highlight the sensitivity of microbial assemblages to anthropogenic interventions and their critical role in shaping ecosystem resilience under chemical stress.

The present Research Topic reinforces the complexity of microbial-fungal symbioses across a wide range of ecological and applied contexts, from plant-associated microbiomes to soil and insect systems. The published studies demonstrate that inter-kingdom interactions are central to regulating diverse functions, including plant fitness, metabolic dynamics, ecosystem resilience, and responses to environmental and anthropogenic pressures. The findings presented here provide new conceptual and methodological insights for understanding how microbial consortia can be explored to improve agricultural sustainability and ecosystem restoration. Future research should further elucidate the molecular and ecological basis regulating these interactions, with a focus on translating these findings into scalable biotechnological applications.
